# The Burden of Hypoxic Respiratory Failure in Preterm and Term/Near-term Infants in the United States 2011-2015

**DOI:** 10.36469/001c.9682

**Published:** 2019-06-19

**Authors:** Shivani Pandya, Onur Baser, George J. Wan, Belinda Lovelace, Jim Potenziano, An T. Pham, Xingyue Huang, Li Wang

**Affiliations:** 1 STATinMED Research; 2 Internal Medicine University of Michigan; 3 Mallinckrodt Pharmaceuticals; 4 School of Pharmacy University of California San Francisco

**Keywords:** health care costs, nitric oxide, meconium aspiration syndrome, pulmonary, hypertension, newborn, infant

## Abstract

**Objectives:** This study quantified the burden of hypoxic respiratory failure (HRF)/persistent pulmonary hypertension of newborn (PPHN) in preterm and term/near-term infants (T/NTs) by examining health care resource utilization (HRU) and charges in the United States.

**Methods:** Preterms and T/NTs (≤34 and >34 weeks of gestation, respectively) having HRF/PPHN, with/without meconium aspiration in inpatient setting from January 1, 2011-October 31, 2015 were identified from the Vizient database (first hospitalization=index hospitalization). Comorbidities, treatments, HRU, and charges during index hospitalization were evaluated among preterms and T/NTs with HRF/PPHN. Logistic regression was performed to evaluate mortality-related factors.

**Results:** This retrospective study included 504 preterms and 414 T/NTs with HRF/PPHN. Preterms were more likely to have respiratory distress syndrome, neonatal jaundice, and anemia of prematurity than T/NTs. Preterms had significantly longer inpatient stays (54.1 vs 29.0 days), time in a neonatal intensive care unit (34.1 vs 17.5 days), time on ventilation (4.7 vs 2.2 days), and higher total hospitalization charges ($613,350 vs $422,558) (all P<0.001). Similar rates were observed for use of antibiotics (96.2% vs 95.4%), sildenafil (9.5% vs 8.2%), or inhaled nitric oxide (93.8% vs 94.2%). Preterms had a significantly higher likelihood of mortality than T/NTs (odds ratio: 3.6, 95% confidence interval: 2.3-5.0).

**Conclusions:** The findings of more severe comorbidities, higher HRU, hospitalization charges, and mortality in preterms than in T/NTs underscore the significant clinical and economic burden of HRF/PPHN among infants. The results show significant unmet medical need; further research is warranted to determine new treatments and real-world evidence for improved patient outcomes.

## Introduction

Neonatal hypoxic respiratory failure (HRF) is a severe respiratory illness that affects 2% of all live births and is responsible for >33% of all neonatal mortality.^1^ In the United States alone, the annual number of term and late pre-term newborns with HRF is estimated at 80,000.^1^ About 15% of term infants and 29% of late-preterm infants admitted in a neonatal intensive care unit (NICU) develop respiratory morbidity.^2^ Respiratory failure in these newborns is often associated with persistent pulmonary hypertension of the newborn (PPHN), which contributes to hypoxemia.^3^ PPHN further complicates the course of respiratory failure in these infants and is a source of increased burden associated with health care costs as well as the indirect burden to these patients’ families and caregivers.^3,4^

The etiology of neonatal HRF includes aspiration of meconium, respiratory distress syndrome, pneumonia, congenital diaphragmatic hernia, and oligohydramnios.^5^ Additionally, exposure to specific drugs—including non-steroidal anti-inflammatory drugs (NSAIDs) and anti-depressants—during pregnancy is associated with increased prevalence of PPHN among neonates.^6^ Each condition may cause an increase in pulmonary shunting, limited lung volume, decreased compliance, or a combination of all three of these pathogeneses, resulting in hypoxemia, hypercarbia, and acidosis, all of which increase the morbidity in neonates with HRF.^7^

The standard treatment for neonatal HRF includes conventional mechanical ventilation, respiratory alkalosis, ionotropic support, systemic infusion of vasodilators, neuromuscular blockade, and sedation.^5^ Traditional neonatal HRF therapies, including mechanical ventilation, have failed to reduce the mortality rate and often resulted in the use of more invasive procedures, including extracorporeal membrane oxygenation (ECMO).^3,4,7^ New and advanced treatments including administration of exogenous surfactant, inhaled nitric oxide, high-frequency ventilation, and ECMO have improved survival rates among neonates with HRF.^8–11^

Neonates with HRF may require multiple and concurrent therapies, which maybe suggestive of extensive use of health care resources with potential implications on economic burden in these patients. To date, there is a lack of real-world evidence quantifying the burden of hospitalization in managing these patients. The aim of this retrospective observational study is to understand and quantify the clinical and economic burden of hospitalization in infants with HRF/PPHN. In an attempt to evaluate this burden, we conducted a retrospective study using a large hospital database to describe the clinical characteristics, health care resource utilization (HRU), costs and charges among preterm and term and near-term (T/NT) infants with HRF/PPHN in the United States.

## Methods

### Data source

This was a retrospective cohort study using the Vizient (formerly MedAssets) Health System Database from January 1, 2011 through December 31, 2015. Vizient is an administrative patient-level database which includes inpatient and hospital-based outpatient information from more than 400 hospitals across 42 US states (59% Southern, 17% Western, 13% Midwestern, 12% Northeastern region).^12^ Hospitals included large and small facilities in urban (87%) and rural (13%) locations. Inpatient and outpatient data are submitted by ~98% of providers, and data are updated twice monthly with a 30-to-45-day lag from month-end. The data include patient demographic information (age, sex, ZIP code, admit source, admit type, discharge status, etc), procedural and diagnosis codes with procedure date, detailed insurance plan with financial class information, total patient charges, patient-level costs, and hospital reimbursement. Ambulatory Payment Classification) as well as Charlson and Elixhauser comorbidity index methodologies.

The data also include information about various clinical grouping methodologies (Medicare Severity- Diagnosis Related Group; Ambulatory Payment Classification) as well as Charlson and Elixhauser comorbidity index methodologies.

### Study population

The eligible study population included preterm (≤34 weeks of gestation; International Classification of Diseases, 9th Revision, Clinical Modification [ICD-9 CM] codes: 765.21, 765.22, 765.23, 765.24, 765.25, 765.26, 765.27; ICD-10-CM codes: P07.21, P07.22, P07.23, P07.24, P07.25, P07.26, P07.31, P07.32, P07.33, P07.34, P07.35, P07.36, P07.37) or T/NT infants (>34 weeks of gestation; ICD-9-CM codes: 765.28, 765.29; ICD-10-CM codes: P07.38, P07.39) who had a diagnosis of HRF/PPHN (idiopathic PPHN [ICD- 9-CM code: 747.83; ICD-10-CM code: P29.3] with or without meconium aspiration [ICD-9-CM codes 770.11, 770.12; ICD-10-CM codes: P24.00, P24.01]) in the inpatient setting during the identification period (January 1, 2011 to October 31, 2015).

The first hospitalization (from admission date to discharge date) during this period which included an HRF/PPHN diagnosis was defined as the index hospitalization.

Preterm and T/NT infants diagnosed with HRF/PPHN during an inpatient visit within the identification period were further stratified as preterm infants with HRF/PPHN and T/NT infants with HRF/PPHN.

### Study variables

Patient characteristics including sex, most common comorbid conditions, and provider characteristics (US region, facility bed number, teaching hospital status, and urban/rural location) were examined for the index hospitalization period. To depict clinical care for hospitalized infants with HRF/PPHN, clinical procedure and treatments (ie, antibiotics, surfactants, inhaled nitric oxide, and sildenafil) were also examined. Treatments were identified based on the patient-level charge description using key words (eg, to identify the use of surfactants, the following key words were used: poractant alfa, Curosurf^®^, beractant, Survanta^®^, etc.), as the pharmacy file with National Drug Codes (NDCs) was not available.

The primary outcomes of interest during the index hospitalization included HRU, total hospitalization costs, and charges. Charges represent the amount billed for health care services rendered by providers, whereas costs represent the amount actually paid for these services. Total costs and charges were also reported according to teaching hospital status and whether the patient died during the index hospitalization. HRU included average length of stay (LOS), NICU use, time in the NICU, ventilation use (non-invasive ventilation [Current Procedural Terminology (CPT) code: 94660; ICD-9/10 procedure codes: 93.90, 5A09357, 5A09457, 5A09557]); invasive mechanical ventilation (CPT code: 31500; ICD-9/10 procedure codes: 96.04, 96.7x, 0BH17EZ, 0BH18EZ, 5A1935Z, 5A1945Z, 5A1955Z), time on ventilation, and ECMO (ICD-9/10 procedure codes: 39.65, 5A15223; CPT codes: 33960, 33961, 36822). Additionally, LOS was reported according to teaching hospital status and patient gestational age. In-hospital mortality rates during index hospitalization were also evaluated and reported.

### Statistical analysis

All study variables including demographics, provider characteristics, and outcomes were analyzed descriptively among the overall infant population and among preterm and T/NT infants in the study sample. Means and standard deviations were provided for continuous variables. Numbers and percentages were provided for categorical variables. Statistical tests of significance (chi-square tests for categorical variables and student t-tests for continuous variables) were conducted to assess the differences between the cohorts. Additionally, for the economic outcomes, the p-values were obtained by using the student t-test in the log-transformed costs. A logistic regression model was performed to assess factors associated with mortality. Patient characteristics such as sex, comorbidities, and provider characteristics—including US region and teaching hospital status, and HRU (average LOS, NICU use, ventilation use, and treatments)—were included as independent variables. All analyses were conducted using SAS statistical software (Version 9.3).

## Results

### Demographic and clinical characteristics

The study included a total of 918 infants with HRF/PPHN during index hospitalization, including 504 preterm (55%) and 414 T/NT (45%). Additionally, the prevalence of HRF/PPHN was 50.1% and 66.9% among preterm and T/NT infants in the NICU, respectively (data not shown). Overall, most infants were male (59.0%) and had respiratory distress syndrome (53.7%), followed by patent ductus arteriosus (50.1%) and neonatal jaundice due to preterm delivery (41.7%) (Table 1).

Hospital providers were geographically distributed across the United States, with more than half of the infants treated at hospitals in the Southern US region (66.0%) followed by 13.4% treated at hospitals in the Western region, 10.4% treated at hospitals in the Northeastern region, and 10.2% treated at hospitals in the Midwestern US region (Table 1). Additionally, a majority of the infants were treated at hospitals located in urban locations (95.1%) with only a small proportion treated in rural hospitals (0.3%). Also, about half of the infants were treated at a major teaching hospital (50.2%), 21.8% were treated at minor-teaching hospital, and 23.4% were treated at non-teaching hospital. Most infants were treated at large hospitals, with 52.4% infants treated at hospitals with ≥500 beds and 26.0% infants treated at hospitals with 300-499 beds. Among preterm infants with HRF/PPHN, a majority were born at a gestational age of 27-28 weeks (29.6%) followed by 33-34 weeks (27.6%), 29-30 weeks (21.6%) and 31-32 weeks (21.6%). Most T/NT infants with HRF/PPHN were born at the gestational age of 35-36 weeks (69.3%) followed by ≥37 weeks (30.7%) (data not shown).

There were no significant differences associated with patient sex between preterm and T/NT infants with HRF/PPHN (male: 59.3% vs 58.7%) (Table 1). Compared with T/NT infants, preterm infants with HRF/ PPHN were significantly more likely to have respiratory distress syndrome (69.4% vs 34.5%, P<0.0001), neonatal jaundice due to preterm delivery (55.4% vs 25.1%, P<0.0001), and anemia of prematurity (49.2% vs 17.9%, P<0.0001), but less likely to have respiratory failure of newborn (22.0% vs 37.9%, P<0.0001). Additional demographic and clinical information for preterm and T/NT infants with HRF/PPHN can be found in Table 1.

**Table 1.** Socio-demographic and Provider Characteristics Among Infants diagnosed with HRF/PPHN

**Demographic Characteristics**	**Total Infants with HRF/PPHN (n=918)**	**Infants with HRF/PPHN**		
		**Preterm infants (n=504)**	**T/NT infants (n=414)**	**P-value**
**Sex, n (%)**				
Male	542 (59.0)	299 (59.3)	243 (58.7)	0.8469
Female	375 (40.9)	205 (40.7)	170 (41.4)	0.9052
Unknown	1 (0.1)	0 (0.0)	1 (0.2)	0.2696
**Clinical Characteristics, n (%)**				
Single liveborn in-hospital born with Cesarean section	360 (39.2)	211 (41.9)	149 (36.0)	0.0697
Respiratory distress syndrome	493 (53.7)	350 (69.4)	143 (34.5)	<0.0001
Patent ductus arteriosus	460 (50.1)	265 (52.6)	195 (47.1)	0.0986
Neonatal jaundice due to preterm delivery	383 (41.7)	279 (55.4)	104 (25.1)	<0.0001
Septicemia (sepsis) of newborn	371 (40.4)	206 (40.9)	165 (39.9)	0.7545
Secundum atrial septal defect	380 (41.4)	202 (40.1)	178 (43.0)	0.3721
Other specific conditions in perinatal period	326 (35.5)	171 (33.9)	155 (37.4)	0.2687
Anemia of prematurity	322 (35.1)	248 (49.2)	74 (17.9)	<0.0001
Respiratory failure of newborn	268 (29.2)	111 (22.0)	157 (37.9)	<0.0001
Neonatal thrombocytopenia	265 (28.9)	155 (30.8)	110 (26.6)	0.1639
**Provider Characteristics**				
**US Geographic region, n (%)**				
Northeastern region	95 (10.4)	64 (12.7)	31 (7.5)	0.0099
Midwestern region	94 (10.2)	45 (8.9)	49 (11.8)	0.1483
Southern region	606 (66.0)	334 (66.3)	272 (65.7)	0.8562
Western region	123 (13.4)	61 (12.1)	62 (15.0)	0.2036
**Bed size group, n (%)**				
<100 beds	22 (2.4)	14 (2.8)	8 (1.9)	0.4046
100-199 beds	81 (8.8)	42 (8.3)	39 (9.4)	0.5634
200-299 beds	53 (5.8)	36 (7.1)	17 (4.1)	0.0497
300-499 beds	239 (26.0)	125 (24.8)	114 (27.5)	0.3475
≥500 beds	481 (52.4)	263 (52.2)	218 (52.7)	0.8861
Unknown	42 (4.6)	24 (4.8)	18 (4.3)	0.7651
**Teaching status, n (%)**				
Major teaching	461 (50.2)	244 (48.4)	217 (52.4)	0.2275
Non-teaching	215 (23.4)	136 (27.0)	79 (19.1)	0.0049
Minor teaching	200 (21.8)	100 (19.8)	100 (24.2)	0.1152
Unknown	42 (4.6)	24 (4.8)	18 (4.3)	0.7651
**Urban/rural status, n (%)**				
Urban	873 (95.1)	480 (95.2)	393 (94.9)	0.8283
Rural	3 (0.3)	0 (0.0)	3 (0.7)	0.0556
Unknown	42 (4.6)	24 (4.8)	18 (4.3)	0.7651

HRF: hypoxic respiratory failure; PPHN: persistent pulmonary hypertension of newborn; T/NT: term/near term

### Treatment patterns during index hospitalization

Most infants with HRF/PPHN were treated with antibiotics (95.9%), inhaled nitric oxide (94%) and surfactants (59.3%) (Table 2). Nearly 95% of infants with HRF/PPHN were treated with invasive or non-invasive mechanical ventilation, with an average time on ventilation of 3.6 days; 5.0% were treated with ECMO. Similar rates were observed between preterm and T/NT infants in the use of antibiotics (96.2% vs 95.4%), sildenafil (9.5% vs 8.2%), or inhaled nitric oxide (93.8% vs 94.2%). A clinically meaningful and significantly higher proportion of preterm infants were treated with surfactants (71.2% vs 44.7%, P<0.0001), and fewer preterm infants were treated with ECMO (2.0% vs 8.7%, P<0.0001) as compared to T/NT infants. Additionally, preterm infants had significantly longer time on ventilation (4.7 vs 2.2 days; P<0.0001) and time on inhaled nitric oxide (8.2 vs 5.9 days, P=0.0017) as compared to T/NT infants with HRF/PPHN. Overall, the inpatient mortality rate among infants with HRF/PPHN was 26.1%, with a significantly higher mortality rate (30.2% vs 21.3%, P=0.0023) in preterm infants as compared to T/NT infants with HRF/ PPHN.

**Table 2.** Procedures and Treatments Among Infants diagnosed with HRF/PPHN

**Procedures**	**Total Infants with HRF/PPHN (n=918)**	**Infants with HRF/PPHN**		
		**Preterm infants (n=504)**	**T/NT infants (n=414)**	**P-value**
**Ventilation use, n (%)**				
Overall	865 (94.2)	474 (94.1)	391 (94.4)	0.7976
Time on ventilation (days), mean (SD)	3.6 (7.7)	4.7 (10.0)	2.2 (2.5)	<0.0001
Non-invasive ventilation	334 (36.4)	188 (37.3)	146 (35.3)	0.5235
Invasive mechanical ventilation	836 (91.1)	461 (91.5)	375 (90.6)	0.6386
**ECMO, n (%)**	46 (5.0)	10 (2.0)	36 (8.7)	<0.0001
**Treatments, n (%)**				
Antibiotics	880 (95.9)	485 (96.2)	395 (95.4)	0.5351
Surfactants	544 (59.3)	359 (71.2)	185 (44.7)	<0.0001
Sildenafil	82 (8.9)	48 (9.5)	34 (8.2)	0.4882
Inhaled nitric oxide	863 (94.0)	473 (93.8)	390 (94.2)	0.8222
Time on inhaled nitric oxide (days), mean (SD)	7.2 (11.2)	8.2 (12.5)	5.9 (9.1)	0.0017
**Mortality rate, n (%)**	240 (26.1)	152 (30.2)	88 (21.3)	0.0023

ECMO: Extracorporeal membrane oxygenation; HRF: hypoxic respiratory failure; PPHN: persistent pulmonary hypertension of newborn; SD: standard deviation; T/NT: term/near term

### HRU, charges and costs during index hospitalization

**Figure 1.**
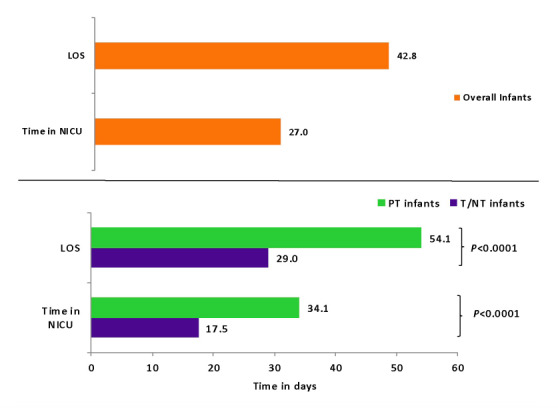
Health Care Resource Utilization Among Infants diagnosed with HRF/PPHN LOS: length of stay; NICU: neonatal intensive care unit; T/NT: term/near term; PT: pre-term

Among all infants with HRF/PPHN, the average inpatient LOS was 42.8 days (median: 26 days), and 61.4% had NICU use with an average duration of 27 days (median: 15 days) in the NICU (Figure 1). Compared to T/NT infants, preterm infants had significantly longer inpatient LOS (54.1 vs 29.0, P<0.0001) and time in the NICU (34.1 vs 17.5, P<0.0001). The median inpatient LOS was 38 days and 20 days, and the median time in NICU was 21 days and 10 days in the preterm and T/NT infants, respectively (data not shown). Additionally, our results showed that the LOS among infants with HRF/PPHN also varied according to teaching hospital status, with the mean LOS being longest among infants treated at a major teaching hospital (46.0 days), followed by those treated at a minor teaching hospital (37.0 days) and non-teaching hospital (36.1 days) (E-Table 1). Among preterm infants with HRF/PPHN, the mean LOS was longest for those treated at a major teaching hospital (57.7 days) followed by those treated at a non-teaching hospital (47.4 days) and a minor teaching hospital (45.7 days). Also, the mean LOS was longest for T/NT infants treated at a major teaching hospital (32.9 days), followed by those treated at a minor teaching hospital (28.2 days) and non-teaching hospital (16.6 days). Additionally, our results showed that LOS was longest among infants born at a gestational age of 27-28 weeks (76.2 days), followed by those born at 29-30 (55.6 days), 31-32 (48.6 days), 33-34 (34.4 days), 35-36 (27.3 days), and ≥37 weeks of gestation (32.8 days) (data not shown).

**Figure 2.**
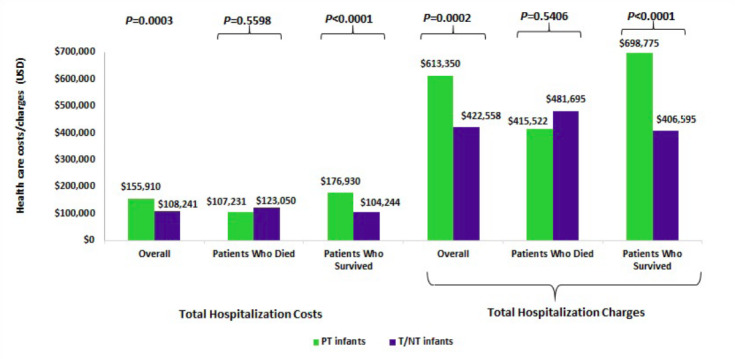
Economic Outcomes Among Preterm and Term/Near-term Infants diagnosed with HRF/PPHN PT: pre-term; T/NT: term/near term Note: Charges represent the amount that has been billed for the services by the providers, whereas costs represent the actual amount paid for these services.

The total hospitalization costs and charges for infants with HRF/PPHN were $134,412 (median: $67,479) and $527,306 (median: $270,954), respectively. Consistent with hospital LOS, preterm infants incurred higher total hospitalization costs ($155,910 vs $108,241, P<0.0001) and charges ($613,350 vs $422,558, P<0.0001) than T/NT infants (Figure 2). Similarly, the median hospitalization costs were $91,515 and $54,783, and the median hospitalization charges were $364,723 and $211,023 in the preterm and T/NT infants, respectively. However, it was observed that preterm infants had significantly lower total hospitalization costs per day ($3,619 vs $4,606, P<0.0001) and lower hospitalization charges per day ($14,260 vs $18,071, P=0.0005) when compared to T/NT infants (data not shown). The average cost-to-charge ratio was 0.26 in the overall population and similar between the preterm and T/NT infants (0.26 vs. 0.26, P=0.0928). Subgroup analysis among infants who survived revealed that preterm infants incurred significantly higher average total hospitalization costs ($176,930 vs $104,244, P<0.0001) and charges ($698,775 vs $406,595, P<0.0001) than T/NT infants.

Consistent with hospital LOS, total hospitalization costs and charges also varied by teaching hospital status. The highest costs and charges were incurred by infants treated at a minor teaching hospital ($158,223; $671,377) followed by those treated at a major teaching hospital ($131,014; $518,802) and a non-teaching hospital ($100,979; $393,392) respectively (E-Table 1). Among preterm infants with HRF/PPHN, the total costs and charges were highest among those treated at a minor teaching hospital ($156,608; $667,990) followed by those treated at a major teaching hospital ($156,397; $633,137) and non-teaching hospital ($132,601; $512,394) respectively. Additionally, the total costs and charges were the highest among T/ NT infants treated at a minor teaching hospital ($159,837; $674,854) followed by those treated at a major teaching hospital ($102,472; $390,242) and non-teaching hospital ($46,450; $188,527) respectively.

### Factors associated with mortality in preterm and T/NT infants with HRF/PPHN

**Figure 3.**
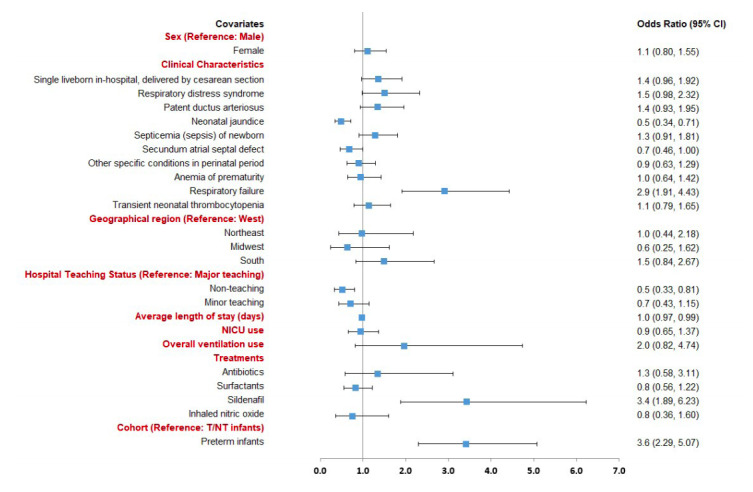
Factors Associated with Mortality Among Preterm and Term/Near-term Infants diagnosed with HRF/PPHN CI: confidence interval; NICU: neonatal intensive care unit; T/NT: term/near-term

Preterm or T/NT infants with evidence of respiratory failure (odds ratio [OR]: 2.9, 95% confidence interval [CI]: 1.9-4.4) or treatment with sildenafil (OR: 3.4, 95% CI: 1.9-6.2) had a significantly increased likelihood of mortality. Those with neonatal jaundice due to preterm delivery (OR: 0.5, 95% CI: 0.3-0.7), secundum atrial septal defect (OR: 0.7, 95% CI: 0.5-1.0), longer hospital LOS (OR: 0.98, 95% CI: 0.97-0.99), and those born in a non-teaching hospital (OR: 0.5, 95% CI: 0.3-0.8) had a significantly lower likelihood of mortality (Figure 3). Additionally, preterm infants (OR: 3.6, 95% CI: 2.3-5.0) had a significantly higher likelihood of mortality compared to T/NT infants with HRF/PPHN after adjusting for the patient characteristics.

## Discussion

To our knowledge, this is the first real-world study to evaluate the comorbidities, treatments, HRU, and health care costs among preterm and T/NT infants with HRF/PPHN using a large US hospital database. Given the higher mortality rate and the need for prolonged cardiopulmonary support in the surviving neonates,^1,13^ it is important to evaluate the clinical and economic burden of HRF/PPHN to successfully aid health care decision making, thereby reducing the burden in this vulnerable patient population.

Our results showed that preterm infants diagnosed with HRF/PPHN were more likely to have evidence of respiratory distress syndrome and associated jaundice, which is in agreement with previous studies that observed respiratory and gastrointestinal infections as common preterm-associated comorbidities.^14,15^ In general, preterm infants may have more adverse birth outcomes associated with the shorter gestation period.^14,15^ Due to the shortened gestation period (≤34 weeks), fetal development may be incomplete in preterm infants, increasing the likelihood of a variety of complications. In fact, fetal development is considered a strong indicator of comorbidities and neonatal mortality.^15^ As expected, our results showed that after adjusting for patient characteristics, the likelihood of mortality was ~4 times higher among preterm compared to T/NT infants, which could be justified from the higher risk profile of preterm infants due to their shortened gestational age.^15^ High mortality among preterm infants in our study can be explained by the increased prevalence of comorbidities including respiratory distress syndrome, asphyxia, and sepsis caused by incomplete fetal development.^16,17^

Considering that HRF/PPHN can vary from mild hypoxemia with minimal respiratory distress to severe hypoxemia and cardiopulmonary instability, the overall goal of treatment is to improve oxygen levels in the blood with various vasodilators including inhaled nitric oxide, magnesium sulfate, adenosine, and phosphodiesterase inhibitors.^18,19^ Additionally, as infection/sepsis was considered the major underlying cause of HRF/PPHN in infants, supportive therapy with antibiotics is recommended.^18–20^ Consistent with these treatment strategies, our results showed that the majority of preterm or T/NT infants had treatment with antibiotics (96%) and inhaled nitric oxide (94%). Despite the recommendations against the use of inhaled nitric oxide in preterm infants with HRF,^21–23^ our study highlights a higher prevalence of off-label use of inhaled nitric oxide among preterm infants with HRF/PPHN. Suzuki *et al* showed that inhaled nitric oxide has helped improve oxygenation in both preterm and T/NT infants with HRF/ PPHN, thereby reducing the need for ECMO, which is a complicated procedure used for infants who fail to respond to medical treatment.^24^ ECMO is also associated with serious adverse effects such as intracranial hemorrhage and ligation of common carotid artery, potentially adding to higher HRU and costs among infants with HRF/PPHN.^25,26^ Despite the use of various vasodilators, HRF/PPHN remains a main source of morbidity and mortality in preterm and T/NT infants, which is also evident from our study showing an overall mortality rate of 26%.^18^ Although the mortality of T/NT infants observed in our study (21%) was ~3 times higher than that reported in a recent review of statewide statistics in California by Steurer *et al* (7.6%),^20^ the overall mortality rate in our study is consistent with the findings from other studies that have estimated the mortality of newborns with PPHN across various centers in the United States at 4-33%.^18,27^

The results of our study showed that, due to the higher prevalence of comorbidities and the need for highly specialized treatments, the economic burden on preterm and T/NT infants with neonatal HRF/ PPHN, including HRU and cost of care, is substantial. More specifically, the economic burden was ~1.5 times higher among preterm infants as compared to T/NT infants with HRF/PPHN. Our results are in agreement with a retrospective study conducted by Hall *et al* in which the excess costs attributable to prematurity in hospitals of Hamilton County, Ohio was estimated as $93 million.^28^ Additionally, Hall *et al* observed that the incremental cost burden was highest in preterm infants born at 27 weeks. However, Hall *et al* estimated the costs associated with preterm birth in general and did not focus on any specific comorbidity. Also, the lower hospitalization costs per day in the preterm infants further emphasize that the total costs in preterm infants were driven by the longer LOS in the hospital and NICU. Additionally, the results of our study showed that the health care costs and charges were higher among infants born in a teaching hospital, which is consistent with a study conducted by Hsu *et al*.^29^ Hsu *et al* added that the higher costs related to teaching hospitals may have been attributed to the increased number of procedures, which may have impacted daily HRU, thereby substantially adding to the costs.^29^ However, whether these higher costs in teaching hospitals are offset by improved clinical outcomes or lower mortality rates remains debatable and requires further and more extensive research. The results of our study highlight the substantial burden and the unmet medical need of HRF/PPHN among newborn infants. This study findings may provide insight for further research to explore most appropriate pathways to optimize treatment paradigms for patients with HRF/PPHN and ultimately improve outcomes and lower the economic burden to the healthcare system.

The findings from our study should be viewed in the context of some study limitations. Our study relied on the patient-level data. While this data is extremely valuable for the efficient and effective examination of health care outcomes, treatment patterns, and costs, they are collected for payment purposes, and not research. The presence of a diagnosis code on a medical claim is not a positive presence of disease and may have been incorrectly coded or included as rule-out criteria rather than the actual disease. Certain clinical and disease-specific parameters are not readily available in this hospital data, which may affect study outcomes. Additionally, treatments were identified based on the charge description due to the absence of a separate pharmacy file including NDC codes in the Vizient database. Due to the retrospective nature of the study, there may be residual confounding due to unobserved clinical or other differences such as confounding by indication affecting treatment decision and outcomes. Also, the generalizability of the study findings may be limited to infants with HRF/PPHN identified by selected respiratory diseases. Additionally, the Vizient health system database has a disproportionate distribution of one geographical region (predominantly American Southern region), which could significantly bias the conclusions; thus, the findings’ generalizability to the entire country may be limited. Therefore, the results of our study should be interpreted with caution.

## Conclusions

The findings of more severe comorbidities, higher HRU, hospitalization charges, and mortality among preterm and T/NTs with HRF/PPHN from the latest US data underscore the significant clinical and economic burden of HRF/PPHN among infants. The results show significant unmet medical need; further research is warranted to determine effective treatment options, which could improve patient outcomes and potentially reduce clinical and economic burden in these infants.

## Declaration of funding

This work was supported by Mallinckrodt Pharmaceuticals.

## Declaration of financial/other relationship

S. Pandya and L. Wang are employees of STATinMED Research, which is a paid consultant to Mallinckrodt Pharmaceuticals. O. Baser has nothing to disclose. G Wan, B Lovelace, J Potenziano, and X Huang are employees of Mallinckrodt Pharmaceuticals, the study sponsor. A. Pham was an employee of Mallinckrodt Pharmaceuticals at the time of the study.

## Author contributions

G Wan, B Lovelace, J Potenziano, and X Huang were involved in the conception and design, S. Pandya, L. Wang and O. Baser were involved in the analysis and interpretation of the data. All authors contributed to the drafting of the paper and offering critical revisions for intellectual content. All authors agree to be accountable for all aspects of the work and approve of the final version for publication.

## Acknowledgements

The authors thank Sujana Borra of STATinMED Research for medical writing assistance.

## Figures and Tables

**Figure:** Supplementary Content
